# Porcine Collagen–Bone Composite Induced Osteoblast Differentiation and Bone Regeneration In Vitro and In Vivo

**DOI:** 10.3390/polym12010093

**Published:** 2020-01-04

**Authors:** Eisner Salamanca, Chia Chen Hsu, Wan Ling Yao, Cheuk Sing Choy, Yu Hwa Pan, Nai-Chia Teng, Wei-Jen Chang

**Affiliations:** 1 School of Dentistry, College of Oral Medicine, Taipei Medical University, Taipei 110, Taiwan; D204103004@tmu.edu.tw (E.S.); m204103002@tmu.edu.tw (C.C.H.); shalom.dc@msa.hinet.net (Y.H.P.); dianaten@tmu.edu.tw (N.-C.T.); 2 Dental Department, Taipei Medical University, Shuang-Ho hospital, Taipei 235, Taiwan; 3 Department of Community Medicine, En Chu Kong Hospital, New Taipei City 237, Taiwan; prof.choy@gmail.com; 4 Yuanpei University of Medical technology, Hsin Chu, Taipei 300, Taiwan; 5 Department of Dentistry, Chang Gung Memorial Hospital, Taipei 106, Taiwan; 6 Graduate Institute of Dental & Craniofacial Science, Chang Gung University, Taoyuan 333, Taiwan; 7 School of Dentistry, College of Medicine, China Medical University, Taichung 404, Taiwan; 8 Dental Department, Taipei Medical University Hospital, Taipei 110, Taiwan

**Keywords:** porcine graft, collagen type I, biological apatite, chemical properties

## Abstract

Due to autogenous bone limitations, some substitute bone grafts were developed. Collagenated porcine graft (CPG) is able to regenerate new bone, although the number of studies is insufficient, highlighting the need for future studies to better understand the biomaterial. In order to understand better CPG′s possible dental guided bone regeneration indications, the aim of this work was to determine CPG′s biological capacity to induce osteoblast differentiation in vitro and guided bone regeneration in vivo, whilst being compared with commercial hydroxyapatite and beta tricalcium phosphate (HA/β-TCP) and porcine graft alone. Cell cytotoxicity (WST-1), alkaline phosphatase activity (ALP), and real-time polymerase chain reaction (qPCR) were assessed in vitro. Critical size defects of New Zealand white rabbits were used for the in vivo part, with critical size defect closures and histological analyses. WST-1 and ALP indicated that CPG directly stimulated a greater proliferation and confluency of cells with osteoblastic differentiation in vitro. Gene sequencing indicated stable bone formation markers, decreased resorption makers, and bone remodeling coupling factors, making the transition from osteoclast to osteoblast expression at the end of seven days. CPG resulted in the highest new bone regeneration by osteoconduction in critical size defects of rabbit calvaria at eight weeks. Nonetheless, all biomaterials achieved nearly complete calvaria defect closure. CPG was found to be osteoconductive, like porcine graft and HA/β-TCP, but with higher new bone formation in critical size defects of rabbit calvaria at eight weeks. CPG can be used for different dental guided bone regeneration procedures; however, further studies are necessary.

## 1. Introduction

In the fields of periodontics, endodontics, implantology, maxillofacial surgery, and orthopedics, performing bone regeneration procedures on a daily basis is a common clinical practice. For these kinds of treatments, the patient′s bone, also known as autogenous bone, is the gold standard and, hence, the first choice. From a macro perspective, autogenous bone is insufficient to cover all bone-grafting procedures due to its limited availability. Over two million bone-grafting procedures are estimated to be performed annually worldwide [1]. In addition, the use of autogenous bone is associated with an 8%–39% risk of major and minor complications [2] related to the donor site harvesting procedure, such as infection, wound drainage, hematomas, reoperation, prolonged pain, sensory loss, and keloids. At a minimum, the patient must undergo a second skin incision, in addition to extended anesthesia time and hospital stay [3]. For these reasons, it is important to have various options available to augment, expand, or substitute autologous bone grafts [4]. Technological evolution and an improved understanding of bone biology led to the development of several bone graft substitutes that are currently available for the treatment of large cancellous voids [5]. Depending on their origin, these substitutes can be classified as allografts, xenografts, and synthetic grafts. All of them provide an osteoconductive or structural framework for bone ingrowth that can be used for bone regeneration while reconstructing significant bone voids [6].

Bone substitutes of natural origin exhibit greater osteoconductivity compared to derivative substitutes [5]. Xenografts are good alternatives because of the unlimited supply of available material and because they eliminate the need for extra procedures to harvest bone. Bovine xenograft was popular in the 1960s but fell into disfavor because of the potential for iatrogenic transmission of prion-related diseases to patients treated with this product [7]. However, the risk declined due to the adoption of appropriate heat treatments to deproteinate bone particles [8,9]. Currently, porcine xenograft is more commonly used mainly because pigs have a genotype close to that of humans [10]. Porcine bone has a similar chemical composition and structure as human bone [4,11,12], and it was reported to be biocompatible and highly osteoconductive in clinical studies. Based on the histomorphometric and osteoblast gene expression profiles, the grafting of corticocancellous porcine bone to heal extraction sockets in humans confirmed these characteristics [13].

Collagen type I is part of the chemical composition and structure of human bone. Previous studies demonstrated that collagen has some superior properties to other materials, including hemostatic functions that allow early wound stabilization, hemostatic properties to attract fibroblasts, and semi-permeability, which facilitates nutrient transfer. The major drawback of collagen is its fast biodegradation due to the enzymatic activity of macrophages and polymorphonuclear leucocytes [14,15]. Furthermore, collagen exhibits elasticity and mechanical toughness, while calcium phosphate exhibits mechanical stiffness to withstand compressive loads [16]. Therefore, it is important to incorporate collagen within bone particle grafts to mimic biological human bone for better bone regeneration outcomes. Multiple studies using collagenated porcine graft (CPG) for different augmentation surgeries showed that new bone formation and implant placement can be safely carried out later [17]. Furthermore, CPG was proven to reduce bone loss when compared to naturally healing sockets [18,19]. Although the available data show good results, the number of studies is insufficient, highlighting the need for future studies to better understand CPG. This work was conducted to determine the biological capacity of CPG for the induction of osteoblast differentiation in vitro and guided bone regeneration in vivo in comparison with commercial hydroxyapatite and beta tricalcium phosphate HA/β-TCP and porcine graft alone. The aim was to gain a better understanding of the material and its possible indications in dental guided bone regeneration procedures.

## 2. Materials and Methods

### 2.1. Graft Materials

**Figure 1:**
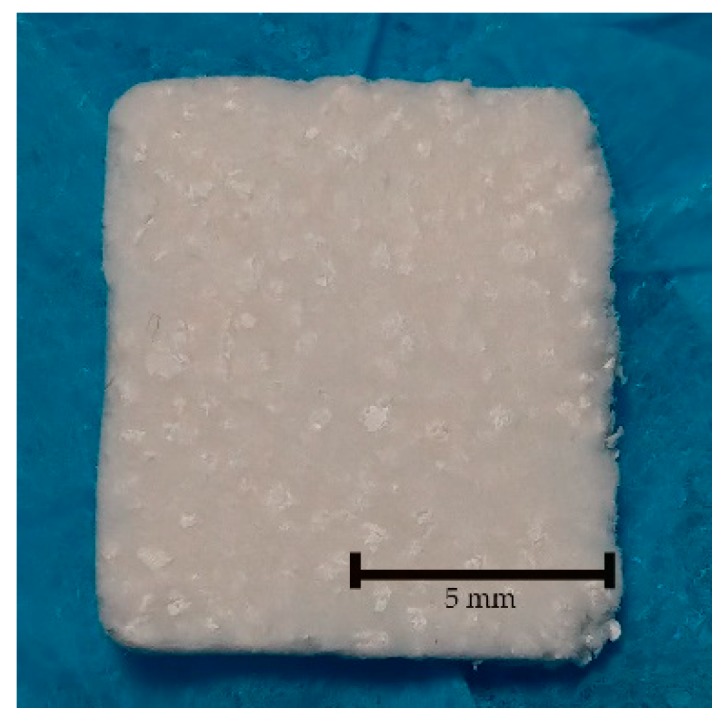
Porcine type I collagen mixed with porcine bone graft at a weight ratio of 30:70. Particles can be seen evenly distributed through the collagen.

CPG is a homogeneous plug consisting of purified porcine type I collagen mixed with porcine bone graft at a weight ratio of 30:70 (Sunmax Biotechnology, Tainan, Taiwan. Figure 1). The preparation process for the material was previously reported in another study performed by the same laboratory [7,20]. Briefly, pig bones were settled in water to remove soft tissue with two different ramp rates until the temperature reached 800 and 1000 °C. Afterward, bone proteins were etched and removed with 0.1–0.5 M hydrochloric acid (HCl) for 10 min. Next, the bones were rinsed, heat-dried, filtered to a particle size of 500–1000 μm, and sterilized using γ-rays. Later, porcine type I collagen was mixed with porcine bone graft, poured into a mold, and allowed to freeze-dry until CPG was formed. HA/β-TCP used was a biphasic ceramic material (MBCP™; Biomatlante, Vigneux de Bretagne, France) consisting of 60% HA and 40% β-TCP with complete interconnected porosity of 70%. The material comprised macropores of >10 µm and micropores of <10 µm in a 2:1 ratio, and it was sintered at temperatures >700 °C [21]. 

### 2.2. Cell Culture and Seeding

MG-63 osteoblast-like cells were purchased from Bioresource Collection and Research Center (BCRC, Hsinchu, Taiwan). The cells were expanded in Dulbecco’s modified Eagle’s medium (DMEM; HyClone, Logan, UT, USA) supplemented with l-glutamine (4 mmol/L), 10% fetal bovine serum, and 1% penicillin–streptomycin (HyClone, Logan, UT, USA) at 37 °C in a humidified atmosphere containing 95% air and 5% CO_2_. The confluent cells were sub-cultured to the next passage using 0.05% trypsin– ethylenediaminetetraacetic acid (EDTA) for up to the fourth passage. Once 90% confluence cell density was reached, the concentration was adjusted to 1 × 10^4^ cells/mL, and the cells were aliquoted into 24-well Petri dishes (Nunclon; Nunc, Roskilde, Denmark). On the same day, the DMEM medium was mixed with CPG, porcine graft, or HA/β-TCP in a 1 g/10 mL concentration. Twenty-four hours later, the medium in the test well was removed and substituted for test media consisting of the previously described DMEM + CPG, porcine graft, or HA/β-TCP. The DMEM medium first described was used for the control wells. The medium was changed every three days for all wells.

### 2.3. Cell Cytotoxicity

Cell cytotoxicity was assessed one, three, and seven days after DMEM + particle grafts were added to the test wells. The test was performed according to the Cell Proliferation Reagent manufacturer′s instructions (WST-1 Kit, Roche Applied Science, Mannheim, Germany). The assay principle is based on the conversion of the tetrazolium salt WST-1 into a colored dye by mitochondrial dehydrogenase enzymes. In brief, the medium from the cells, prepared as previously described, was replaced with 500 µL of fresh medium. Later, the cells were moved to a 96-well microtiter plate (5 × 10^4^ cells/well) in a final volume of 100 µL of culture medium in the absence of any remaining particle grafts. Afterward, the cells were incubated for 24 h, and 10 µL of WST-1 reagent was added to each well, followed by incubation for 4 h in the same standard culture conditions. Next, the plate was placed on a shaker for 1 min to mix the contents. Subsequently, the absorbance of the samples was measured using a Multiskan™ GO Microplate Spectrophotometer (Thermo Fisher Scientific, Waltham, MA, USA) at the optical density (OD) of 420–480 nm with a reference wavelength of 650 nm. The percentage cytotoxicity was calculated from the following equation: % cytotoxicity = (100 × (control − sample))/control [22].

### 2.4. Assessment of Cell Morphology by Fluorescence Microscopy

Image analysis-based cell morphology in two-dimensional (2D) culture was evaluated by staining with phalloidin and with the DNA-binding dye DAPI (4′,6-diamidine-2′-phenylindole dihydrochloride) according to manufacturer′s protocols (D9542 Sigma-Aldrich Deisenhofen, Germany). Staining was performed one, three, and seven days after cell culture. Cells were fixed using 4% paraformaldehyde. Substrates were then washed with buffer to remove loosely bound cells prior to imaging. Morphological results of the cell culture were visualized at one, three, and seven days using an inverted fluorescence microscope (Leica, DFC 7000T, Wetzlar, Germany) with excitation at 488 nm and detection at 530 nm fluorescin diacetate (FDA, green) and 620 nm propidium iodide (PI, red). Visual field was selected randomly [23].

### 2.5. Alkaline Phosphatase Activity

After cell culture and seeding, alkaline phosphatase activity was measured on the first, third, and seventh days. Cells were washed twice with phosphate-buffered saline (PBS). PBS was removed using suction, and 300 μL of Triton X-100 (BioShop, Canada Inc. Burlington, Ontario, Cannada) was added at a concentration of 0.05%. To induce rupture, the cells were subjected to three cycles of 5 min at 37 °C and 5 min at −4 °C, and the samples were later placed into 96-well plates. The alkaline phosphatase (ALP) activity was determined by following the Thermo Scientific 1-Step *p*-nitrophenyl phosphate disodium salt (PNPP) manufacturer’s instructions. PNPP was supplied pre-mixed with substrate buffer and ready to use at room temperature. The 1-Step PNPP was gently mixed. Next, 100 µL of the mixture was added to each well in the 96-well plate and mixed thoroughly by gently agitating the plate, and the 96-well plates were incubated at room temperature for 30 min. To stop the reaction, 50 µL of 3 M NaOH was added and mixed thoroughly by gently agitating the plate. The absorbance of each well was measured at 405 nm using a Multiskan™ GO Microplate Spectrophotometer (Thermo Fisher Scientific, Waltham, MA, USA). The enzymatic activity was normalized to the total protein concentration using bovine serum albumin (Roche, Basel, Switzerland). The measurement of protein was done by using the standard Bradford method (Sigma). The ALP activity was expressed as μm/mg protein/assay time. The comparison was done by plotting the OD intensity [24].

### 2.6. Real-Time Polymerase Chain Reaction (qPCR)

For RNA processing, cell culture and seeding were performed after zero, three, and seven days. Total RNA was extracted using the Novel Total RNA Mini Kit (NovelGene, Molecular Biotech, Taiwan) according to the manufacturer′s instructions. For RNA processing, the cells were trypsinized, harvested, and resuspended in 100 μL of PBS, before being subjected to cell lysis by adding 400 μL of natural rubber and 4 μL of *S*-mercaptoethanol to the sample. RNA binding was later performed with 400 μL of 70% ethanol and centrifuged at 13,000 rpm. Afterward, the sample was washed and eluted with 50 μL of RNase-free water [25,26].

Expression was quantified using qPCR. Gene expression levels were normalized to the expression of the housekeeping gene glyceraldehyde 3-phosphate dehydrogenase (*GAPDH*). Analysis results were expressed as time-course gene changes relative to the cell’s genes cultured in DMEM only, and the calibrator sample representing the amount of transcript was expressed on day zero [27]. Real-time PCR was performed using 2 μL of complementary DNA (cDNA) in a 20-μL reaction volume with the LightCycler® 96 Instrument and application software (Roche Molecular Systems, Inc., Pleasanton, CA, USA), and Fast SYBR^tm^ Green Master Mix (Thermo Fisher Scientific, Vilnius, Lithuania). The temperature profile of the reaction was 95 °C for 10 min, followed by 40 cycles of denaturation at 95 °C for 15 s, annealing at 60 °C for 60 s, and extension at 72 °C for 30 s. Quantification was performed using the delta–delta calculation method. Forward and reverse primer sequences were designed using Primer-BLAST from the United States (US) National Library of Medicine and are listed in Table 1 [28]. 

### 2.7. In Vivo Test

All animal experiments were approved by the Taipei Medical University animal ethics committee and performed following the laboratory animal center guidelines using a protocol previously described by the same laboratory [7,20]. Twenty adult male New Zealand white rabbits (mean age: 12 weeks, mean weight 3.2 kg) were housed in cages at 19 °C and 55% humidity and fed standard rabbit chow and water ad libitum. Anesthesia was administered using an intramuscular injection of Zoletil 50 (50 mg/ml. Vibac Laboratories, Carros, France) at 15 mg/kg into the gluteal region, and surgery was performed on the animals after 10 min of sedation. The calvaria region was then shaved, draped, and sterilized using iodine. Local anesthesia with 1.8 mL of 2% lidocaine with epinephrine at 1/100,000 was injected as a hemostatic in the region. Subsequently, a 2-cm longitudinal midline vertical skin and periosteum incision was made. Calvaria bones were exposed, and four (6 × 3 mm) critical calvaria defects were created bilaterally in the parietal and frontal bones using a low-speed trephine bur with continuous saline cooling (3I Implant Innovation, Palm Beach Gardens, FL, USA). Each defect was filled with a different material: porcine graft, CPG, or HA/β-TCP. Only the control defect was left unfilled. The rabbits were kept in cages under surveillance for the first 24 h and then examined every three days for two weeks and weekly thereafter. The animals (*n* = 5 per group) were sacrificed at two, four, six, and eight weeks after surgery. Euthanasia was performed by CO_2_ asphyxiation 10 min after intramuscular injection of Zoletil 50 (50 mg/mL) at 15 mg/kg into the gluteal region. Subsequently, defects were recovered and prepared for micro-computed tomography (micro-CT) and histological analysis. 

### 2.8. Micro-CT Scanning Cortical Defect Closure

Sample blocks were prepared in formalin and micro-computed tomography (micro-CT) scanning analyses was performed within two weeks using Skyscan 1076 (Skyscan, Antwerp, Belgium). After setting the micro-CT images, coronal images of the upper and lower peripheral areas of the defect were saved in the database, and three-dimensional (3D) morphological analyses were performed for cortical defect closure measurements. Thus, binary selections of samples from the morphometric analyses were made according to grayscale density between units of 20 and 80. The analyses were performed using Skyscan 1076 data-viewer software according to the manufacturer′s instructions.

### 2.9. Histological Analysis

Masson′s trichrome (MT) staining was performed for the detection of new bone formation and the remaining graft material. The harvested samples were decalcified in 10% EDTA for two weeks, embedded in paraffin, and cut from a sagittal perspective using a microtome (Leica RM2145 microtome, Wetzlar, Germany) to obtain the two most central sections (5 µm thick) for each defect [29]. Histomorphometric studies were performed by an experienced investigator blinded to the experiment. For histomorphometric analysis, images of three regions of interest (ROIs), as well as the borders and middle of the defect (2 mm × 2 mm), were captured using a Leica/Aperio ScanScope System. To measure new bone formation and the remaining graft material, ImageJ software (National Institutes of Health; Bethesda, MD, USA) was used [7].

### 2.10. Statistical Analysis

Comparisons were made between the CPG, porcine graft, HA/β-TCP, and control groups at different time periods. A non-parametric one-sample Wilcoxon test was used to identify differences between groups, while a nonparametric Kruskal–Wallis test followed by a Mann–Whitney test was used to identify statistical differences between the different time points. For all resulting parameters, the mean ± standard deviation was used, and statistical significance was set at *p* < 0.05 for all the tests. All data analyses were performed with Microsoft Excel Professional Plus 2016 (Microsoft Software, Redmond, WA, USA).

## 3. Results

### 3.1. Cell Culture and Organization

**Figure 2:**
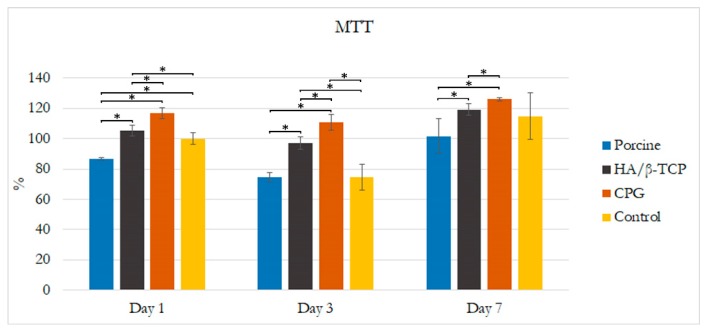
Effects of collagenated porcine graft (CPG), porcine graft, and hydroxyapatite and beta tricalcium phosphate (HA/β-TCP) on MG-63 osteoblast-like cell viability. Cells were incubated from days 0–7. Results are expressed as percentages of control and are the means ± standard error. Statistically significant differences were set at *p* < 0.05 and are indicated by asterisks (*).

**Figure 3:**
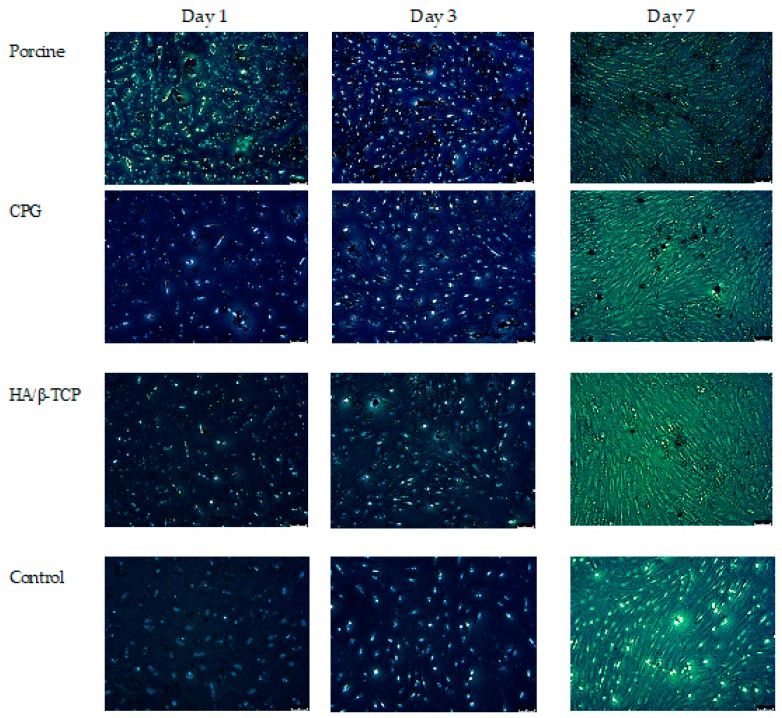
Fluorescence microscopy of MG-63 cells co-cultured with particle graft medium. Changes in MG-63 cell morphology and spreading at one, three, and seven days. (magnification 20×).

The cell cytotoxicity assay results are presented in Figure 2, showing that there was a clear trend of greater proliferation in the DMEM + CPG medium than in the other media at one, three, and seven days. Additionally, at all time points, DMEM combined with CPG or HA/β-TCP was non-toxic and more viable than DMEM + porcine graft and DMEM alone. Cells cultivated in DMEM + porcine graft presented similar toxicity and viability as the control medium without any statistically significant differences in any of the time points. Statistically significant differences (*p* < 0.05) are outlined in Figure 2. The morphology of the incubated MG-63 osteoblast-like cells in all media was similar over seven days when observed with light microscopy. There were no apparent specific changes in the first 24 h, but changes in cell number and confluency were observed after 72 h, with well-attached cells presenting an elongated shape (Figure 3).

### 3.2. Alkaline Phosphatase Assay

**Figure 4:**
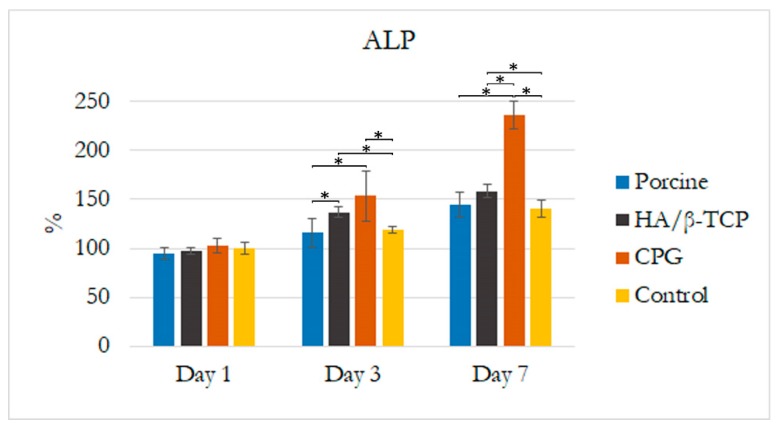
Effects of CPG, porcine graft, and HA/β-TCP on alkaline phosphatase (ALP) activity of MG-63 osteoblast-like cells after seven days. Results are expressed as percentages of control and are the means ± standard error. Statistically significant differences were set at *p* < 0.05 and are indicated by asterisks (*).

The effect of CPG, porcine graft, and HA/β-TCP on cellular differentiation was assessed by measuring ALP activity. As shown in Figure 4, there was a higher increase in ALP expression for cells cultured in DMEM + CPG medium over the other cells on days three and seven (*p* < 0.05). These results strongly suggest that CPG directly stimulates the osteoblastic differentiation of MG-63 osteoblast-like cells in vitro. In addition, DMEM mixed with porcine graft or HA/β-TCP increased the cells’ ALP activity in a time-dependent manner, better than DMEM alone after seven days. Statistically significant differences (*p* < 0.05) are outlined in Figure 4.

### 3.3. Real-Time Polymerase Chain Reaction (qPCR)

To characterize the phenotypes of the differentiated MG-63 and confirm their osteoblast phenotype, the gene expression of cells cultured in DMEM control or combined with CPG, porcine graft, or HA/β-TCP was analyzed by performing qPCR on days three and seven of co-cultivation. The results were compared to those for cells cultured in DMEM only, which was used as the calibrator sample on day zero. The targeted gene markers for bone formation were *ALP* and osteocalcin (*OC*). *CR* and receptor activator of nuclear factor Kappa B (*RANK*) were assigned as markers for bone resorption, and osteoprotegerin (*OPG*), RANK ligand (*RANKL*), and *RANK* were selected as markers for bone remodeling coupling. The expression of *GAPDH* was determined to confirm the usage of similar amounts of RNA for RT-PCR.

**Figure 5:**
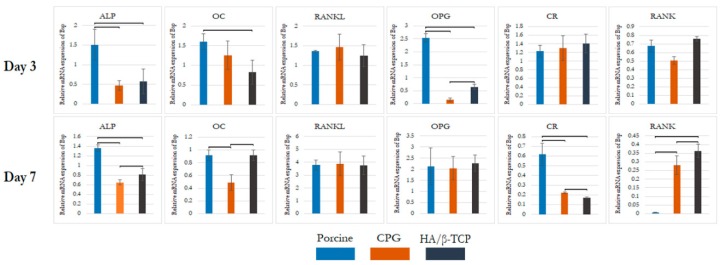
MG-63 gene expression on days three and seven. CPG, porcine graft, and HA/β-TCP in MG-63 osteoblast-like cells induced greater osteoblast gene expression than the control. Statistically significant differences were observed between the groups each day and between days compared to the control, which was established as day zero. Statistically significant differences were set at *p* < 0.05 and are indicated by asterisks (*).

The *ALP* gene expression levels were highest in the porcine graft group on day three and were nearly unaltered by day seven. The *ALP* levels in the CPG and HA/β-TCP groups were consistently better than that in the control group, with an increase in the expression over seven days. The expression levels of *OC* were increased in all biomaterial media on day three compared to that of the control, with the highest increase observed in the porcine graft group. On day seven, *OC* exhibited a reduction in the porcine graft and CPG groups, with no major change in the HA/β-TCP group. The expression level of *CR* was about the same for all three test groups, and the levels in all groups were significantly higher than the control level on day three. This changed on day seven, when significantly lower expression of *CR* was observed compared to that of the control, as well as a significantly lower expression with CPG and HA/β-TCP. The porcine graft group showed reduced *CR* expression on day seven but still had higher expression than the control group, with no significant differences between the two groups (Figure 5).

When the groups were compared at each time point, a similar trend was observed for the *RANKL* expression. On day three, all groups had higher *RANKL* expression than the control, with nearly the same levels (*p* < 0.05). On day seven, a sharp increase in *RANKL* level was demonstrated by all groups with the same behavior, with a statistically significant difference compared to the control on day three. In contrast, *RANK* showed a gradual decrease over time. On day three, all groups had similar *RANK* levels with significant difference from the control, followed by a marked reduction on day seven, with all groups having less *RANK* expression than the control group (*p* < 0.05). *OPG* was expressed in the porcine graft group at a higher level with a sharp increase over the rest of the groups on day three. During the same period, the CPG and HA/β-TCP groups had significantly lower and nearly the same expression levels, respectively, compared to the control (*p* < 0.05). A significant difference was observed on day seven, with the CPG and HA/β-TCP groups reaching the same level as the porcine graft group, which showed a similar pattern as on day three. All groups had statistically significant differences compared to the control on day 7 (*p* < 0.05) (Figure 5).

### 3.4. Defect Closure

**Figure 6:**
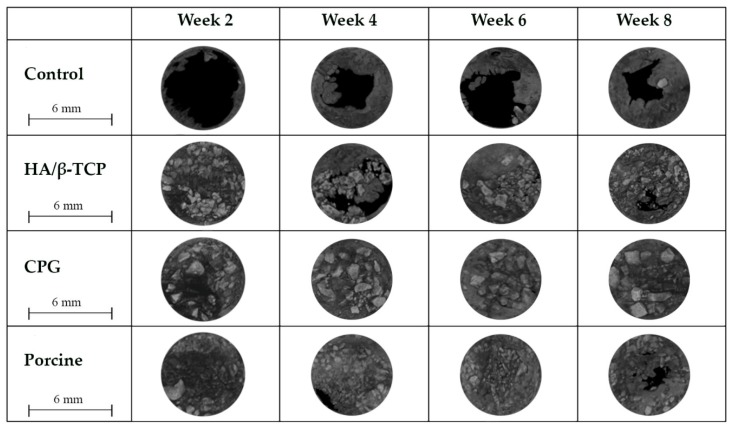
Upper view of critical size defects with non-closure in control defects vs. nearly complete closure with CPG, porcine graft, and HA/β-TCP over eight weeks.

During all study periods, defect closure measured in percentage was found to increase, resulting in the closing of most defects. The control defects did not close entirely and had the least amount of closure among the groups, having only 72.19% ± 24.08% and 64.63% ± 19.04% upper and lower closure overall over eight weeks (Figure 6, Table 2). Due to the incomplete closure of the control defects, they were considered critical size defects. Active treatment of CPG resulted in the best defect closure, with 99.42% ± 1.3% upper and 99.49% ± 1.14% lower closure of the defects (*p* < 0.05). When observed at eight weeks, the HA/β-TCP group (97.19% ± 1.65% upper and 97.73% ± 1.57% lower) was better than the porcine graft group (94.66% ± 5.3% upper and 95.64% ± 3.91% lower), with nearly all the defects closed. Differences between the use of graft materials vs. natural healing were statistically significant (Table 2) in favor of the active biomaterials. 

### 3.5. Histological and Histomorphometric Analyses

**Figure 7:**
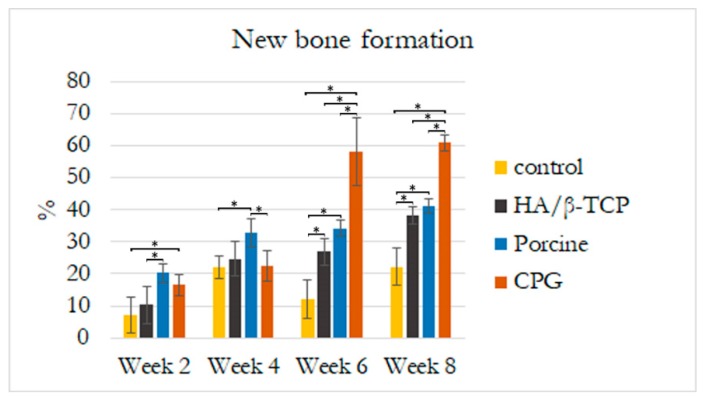
Histological new bone formation percentage at two, four, six, and eight weeks. Bone area was measured in the regions of interest (2 mm × 2 mm) at the borders and center of the most central part of the rabbit calvaria defects. Asterisks (*) indicate statistically significant differences (*p* < 0.05).

**Figure 8:**
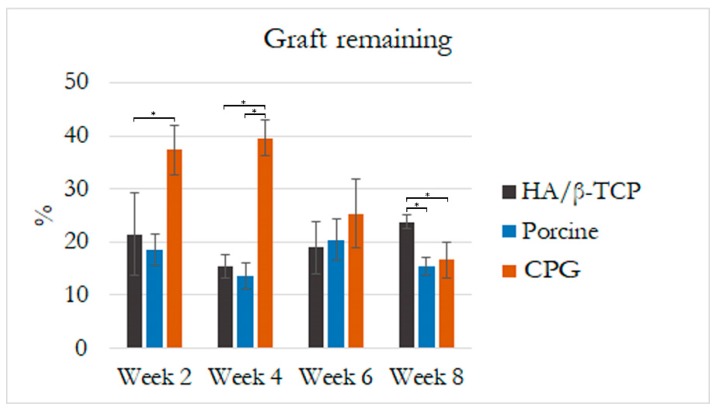
Histological graft remaining percentage at two, four, six, and eight weeks. Graft particle area was measured in the regions of interest (2 mm × 2 mm) at the borders and center of the most central part of the rabbit calvaria defects. Asterisks (*) indicate statistically significant differences with more resorption in CPG at the end of eight weeks (*p* < 0.05).

**Figure 9:**
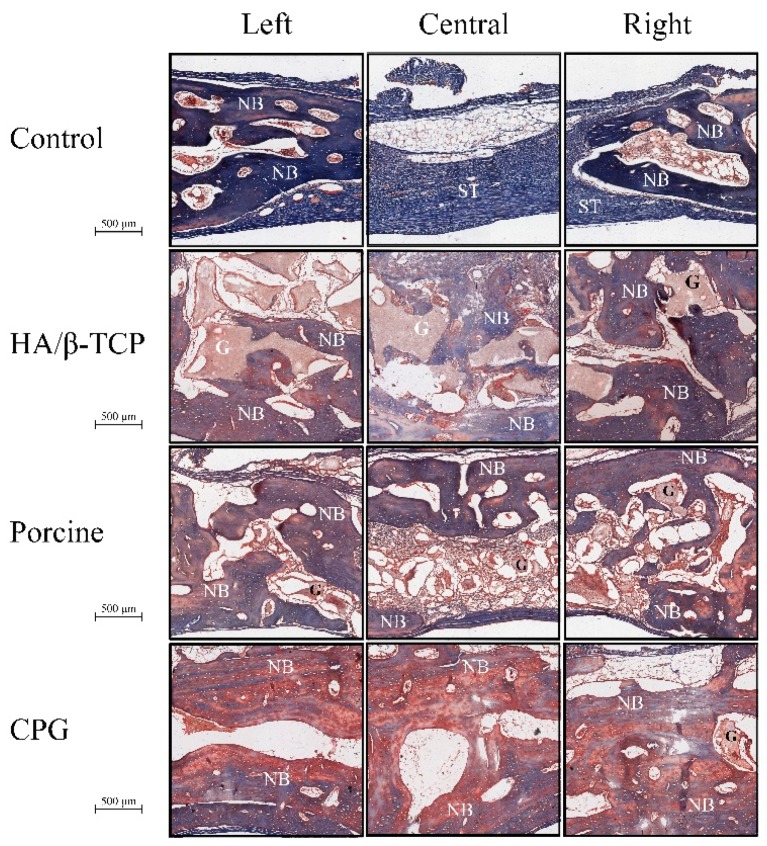
Histological micrographs of mid-sagittal section’s regions of interest at eight weeks. ST: soft tissue, G: graft particles, NB: new bone, OB: old bone.

Histological results revealed that the defect sites exhibited variable degrees of healing between the defects filled with graft materials. All defect sites had a significant difference from the control defects except for the first two weeks when histologic examination revealed the greatest concentrations of immature bone in the borders of all defects. The control defects had the lowest bone formation of 7.07% ± 5.51% followed by HA/β-TCP defects with 10.30% ± 5.65% of new bone and 21.4% ± 7.7% of graft filling the defects. Porcine graft defects with 20.24% ± 3% had the highest amount of new bone formation with only 18.6% ± 3% of the graft filling the defect. The CPG defects had 16.58% ± 3.27% of new bone formation, but double the graft amount filled the defects (37.3% ± 4.7%). In all defects, the discrete presence of some inflammatory multinucleated giant cells and blood capillaries with red blood cells were observed. In addition to this moderate inflammatory response, some fibroblasts were observed around the graft particles in the CPG, porcine graft, and HA/β-TCP defects, indicating a more intense inflammatory response than in the control defects (Figure 7, Figure 8 and Figure 9).

A reduced number of inflammatory cells along with new bone formation and connective tissue were found at four weeks in all defects. Grafted defects presented biomaterial particles surrounded by some demineralized bone particles. In the porcine defects, 32.89% ± 4.37% of new bone was easily distinguishable from the 13.6% ± 2.4% of grafted particles. Similarly, 22.11% ± 3.43% new bone and 22.55% ± 4.76% osteoid formation were observed in the control and CPG defects, with 39.6% ± 3.4% incomplete resorbed graft particles in the CPG defects. HA/β-TCP defects had only 15.4% ± 2.2% of non-reabsorbed particles grafts, but the particles were well integrated and in complete continuity with the 24.72% ± 5.56% of new bone tissue formation (Figure 7, Figure 8 and Figure 9).

In all samples at six weeks, trabecular bone was observed over the entire grafted area, with the highest amount of new bone (57.93% ± 10.51%) on the CPG defects grafted to 25.4% ± 6.5% of the remaining material particles. In the porcine defects, 20.4% ± 3.8% of particles were surrounded by 34.25% ± 2.34% of regenerated thin new bone trabeculae, while others were partially in direct contact with the woven bone. The same characteristics were found within the 26.90% ± 4.13% new bone formation in HA/β-TCP defects, which sometimes presented newly formed bone areas inside the 18.9% ± 4.9% remnant particles (Figure 7, Figure 8 and Figure 9).

At eight weeks, no evidence of foreign-body reaction was observed in any of the samples, and there was no evidence of necrotizing reaction. In many areas of grafted defects, wide osteocyte lacunae and rims of osteoblasts were visible with active deposition of osteoid matrix. This deposition formed 60.92% ± 2.46% of new bone and 16.6% ± 3.3% of particles in CPG defects. In the porcine graft group, the defects consisted of 41.16% ± 2.17% newly formed bone, while the HA/β-TCP defects contained 38.21% ± 2.67% newly formed bone. Furthermore, these new bones were in close and tight contact with 15.4% ± 1.7% and 23.8% ± 1.3% of the particle remnants in the porcine and HA/β-TCP defects, respectively. Similar to the grafted defects, the tissue corresponded in structure and morphology to new bone tissue in the control defects with 22.10% ± 5.81% new bone formation (Figure 7, Figure 8 and Figure 9).

## 4. Discussion

The emphasis of this study was to determine CPG′s biological capacity to induce osteoblast differentiation in vitro and guided bone regeneration in vivo. Gene sequencing indicated stable bone formation markers, decreased resorption makers, and bone remodeling coupling factors, making the transition from osteoclast to osteoblast expression at the end of seven days. CPG resulted in the highest guided bone regeneration in critical size defects of rabbit calvaria at eight weeks, and this was achieved by osteoconduction. CPG was slightly better than or similar to commercial HA/β-TCP and porcine graft, with better in vitro results and new bone formation. Nonetheless, all biomaterials achieved nearly complete calvaria defect closure. CPG was found to be safe, effective, and osteoconductive. Within the characteristics presented in this study, CPG can be indicated for augmentation or reconstructive treatment of alveolar ridge defects, filling in periodontal defects, defects after root resection, apicocectomy, and cystectomy, extraction sockets to enhance preservation of the alveolar ridge, maxillary sinus floor lifting, and periodontal defects in conjunction with products intended for guided tissue regeneration and guided bone regeneration. 

In the cytotoxicity test and fluorescence microscopy, there was a clear trend of greater proliferation and confluency of cells, with good attachment and elongated characteristics in the DMEM + CPG medium than in the other media during the seven days of the test (Figure 2 and Figure 3). These results demonstrated that CPG graft material, due to the presence of collagen, is less toxic and more viable than the other materials and control (Figure 2 and Figure 3). The WST-1 cell proliferation reagent used in the present cytotoxicity assay is similar to the reagent used in the 3-(4,5-dimethylthiazol-2-yl)-2,5-diphenyltetrazolium bromide (MTT) assay in principle, as both measure the metabolic activity of viable cells. However, the WST-1 assay produces water-soluble formazan, eliminating the additional solubilization step. Thus, the WST-1 assay should be considered a more rapid alternative to the MTT assay [22]. ALP activity is essential for bone mineralization and is considered a useful biochemical marker for bone formation [30]. During the same seven-day period when the cytotoxicity test was conducted, MG-63 osteoblast-like cells cultured in DMEM + CPG had higher ALP activity, strongly suggesting that osteoblastic-like cells differentiate by depositing a mineralized extracellular matrix characteristic of bone tissue in vitro (*p* < 0.05) [31] (Figure 3). 

The present study demonstrated that porcine graft increases osteogenic differentiation in the osteoblast-like MG-63 cell line more than the control, evidenced by higher expression levels of bone formation markers including *ALP* and *OC* on days three and seven. Osteogenic differentiation proceeds through different developmental stages characterized by specific markers including early markers such as *ALP* and late markers such as *OC*. Additionally, porcine graft induced a decrease in the expression of the *CR* gene, a bone resorption marker and regulator of calcium metabolism via specific receptors [32]. *RANKL*/*RANK*/*OPG* expression levels on days three and seven indicated that the cells cultured in DMEM + porcine graft had more osteoblast-related gene expression than osteoclasts. CPG and HA/β-TCP, similar to the porcine graft, induced increased osteogenic differentiation in osteoblast-like cells compared to the control. *ALP* and *OC* gene expression was lower in the CPG and HA/β-TCP groups than in the porcine graft group on day three. *CR* gene expression in the CPG and HA/β-TCP groups after seven days approached the level observed in the porcine graft group. The levels of *RANKL*/*RANK*/*OPG* in the CPG and HA/β-TCP groups indicated a slightly higher osteoclast-related gene expression than the levels in the porcine graft group. The *RANKL/RANK/OPG* pathway is important for understanding the signaling between osteoblasts and osteoclasts. Osteoblast cells express *RANKL*, which binds to its receptor, *RANK*, on the surface of osteoclasts and their precursors. This regulates the differentiation of precursors into multinucleated osteoclasts and the activation of osteoclasts. *OPG* is secreted by osteoblasts and osteogenic stromal stem cells and protects the skeleton from excessive bone resorption by binding to *RANKL* and preventing it from interacting with *RANK* [33].

MG63 cells were chosen because they are well characterized as immature osteoblasts, and they were applied as a tool for studying differentiation processes [31,34]. The results for *ALP* gene expression did not correspond to the biochemical analysis results for ALP activity due to the lower cell proliferation found in the cytotoxicity test. This indicated that a higher number of cells cultured with CPG in the ALP biochemical analysis were more able to deposit a mineralized extracellular matrix than cells cultured in porcine and HA/β-TCP media. However, cells cultured in porcine medium had higher osteoblast gene markers, indicating that the porcine graft induced cells into a more mature osteoblast differentiation than CPG and HA/β-TCP. Despite these differences, all three graft materials were superior to the control.

The rabbit calvaria defect animal model was chosen because the rabbit calvarial bone has similar characteristics to those of the jawbone: the intramembranous embryological origin, the presence of two cortical layers separated by cancellous bone, and the physiology of bone repair [35]. All rabbit calvaria defects were considered critical size defects because they failed to heal during the animal′s lifetime in the study, making it an adequate animal model for testing CPG osteoconductivity, biocompatibility, and degradation in comparison to porcine graft and HA/β-TCP. Animal models for bone-grafting studies should be cheap, easy to obtain and handle, and adequate for the creation of large bone cavities [35]. The histomorphometric results in the present study demonstrated defect sites with variable degrees of healing between the defects filled with graft materials, and all of them were significantly different compared to the control defects. At two weeks, the CPG, porcine, and HA/β-TCP defect groups exhibited a moderate inflammatory response, some fibroblasts around the graft particles, and more intense inflammatory responses compared to the control defects. This is in agreement with Figueiredo et al.’s revelation that a xenogeneic graft mixed with collagen type I and an alloplastic material conformed to hydroxyapatite and two secondary phases of α- and β-tricalcium phosphate, similar to the ones used in our study, did not cause severe inflammation in the in vivo inflammatory response test [36]. At eight weeks, wide osteocyte lacunae and active deposition of osteoid matrix on the rims of osteoblasts were observed in many areas of the grafted defects. This deposition was caused by 60.92% ± 2.46% of new bone in the CPG defects and was in close contact with only 16.6% ± 3.3% of the remaining particles. The values were higher than those observed for the porcine and HA/β-TCP defects with 41.16% ± 2.17% and 38.21% ± 2.67% of newly formed bone, respectively. All grafted materials had higher new bone tissue than the control defects, which had only 22.10% ± 5.81% new bone formation (Figure 7, Figure 8 and Figure 9). Similar results were found by Nannmark et al. in a study on rabbit maxillary defects evaluating bone tissue response to prehydrated and collagenated porcine bone graft with or without collagen gel. The group found that both materials showed bone formation directly on the particles with typical osteoblastic seams. Over an eight-week period, the bone area increased and the prehydrated and collagenated porcine bone graft, both mixed with and without collagen gel, was resorbed by osteoclasts. Remodeling was also observed with the formation of osteons within the particles. They concluded that prehydrated and collagenated porcine bone graft exhibited osteoconductive properties and was resorbed with time [37]. Rabbit animal models are sometimes considered the first step before larger animal studies. For particulate porcine bone mix and porcine corticocancellous collagenate prehydrated bone mix for bone regeneration, larger animal studies were performed by surgically creating bone defects around implants in sheep. The findings of this study, in agreement with ours, also revealed new bone formation around the graft particles [38].

Because CPG mimics human bone architecture, it was used in different types of human studies, including successful buccal bone augmentation around immediate implants with and without flap elevation [39]. It was also reported to be non-problematic and predictable in terms of clinical success for a case series of implant placement in fresh extraction sockets and simultaneous osteotome sinus floor elevation [40]. Furthermore, CPG was compared to porcine bone mix collagen with autologous bone and a 50:50 mixture for bone formation in sinus augmentation procedures using histological evaluation after two months. The researchers concluded that collagenated porcine bone alone or in combination with autologous bone is biocompatible and osteoconductive and can be successfully used in sinus augmentation procedures [10].

## 5. Conclusions

CPG had the capacity to induce MG-63 cells into mature osteoblast differentiation in vitro with higher viability and ALP activity than porcine and HA/β-TCP media. The gene sequence in cells cultivated with CPG and HA/β-TCP indicated stable bone formation markers, decreased resorption markers, and bone remodeling coupling factors, making the transition to mature osteoblast expression at the end of seven days. CPG was found to be osteoconductive, similar to porcine graft and HA/β-TCP, but with higher new bone formation in critical size defects of rabbit calvaria after eight weeks. CPG can be used for different dental bone regeneration procedures; however, further studies are necessary.

## Figures and Tables

**Table 1:** Primer sequences for real-time polymerase chain reaction.

Gene Symbol	Forward primer sequence (5′ > 3′)	Reverse primer sequence (5′ > 3′)
*ALP*	AGCCTTCCTGAAAGAGGATTGG	GCCAGTACTTGGGGTCTTTCT
*OC*	TCCTTTGGGGTTTGGCCTAC	CCAGCCTCCAGCACTGTTTA
*RANKL*	ACTGGCCTCTCACCTTTTCTG	AGCCATCCACCATCGCTTTC
*CR*	TTGCTGCCCGCAATTTATGA	TGCTGGCAAGATACTCAGGT
*OPG*	CTGGAACCCCAGAGCGAAAT	GCCTCCTCACACAGGGTAAC
*RANK*	GAAGGTGGACTGGCTACCAC	TTTCCTTCCCCTCCCCAGAA
*GAPDH*	CCTCCTGTTCGACAGTCAGC	CCTAGCCTCCCGGGTTTCTC

**Table 2:** Percentage of calvaria defect closure. CPG—collagenated porcine graft.

	Defect Upper Side				Defect Lower Side			
	2 Weeks	4 Weeks	6 Weeks	8 Weeks	2 Weeks	4 Weeks	6 Weeks	8 Weeks
**Control**	6.25 ± 7.99 *	57.95 ± 17.04 *	56.98 ± 35.34 *	72.19 ± 24.08 *	14.67 ± 11.48 *	55.22 ± 15.8 *	49.28 ± 31.01 *	64.63 ± 19.04 *
**HA/β-TCP**	65.56 ± 20.66	84.22 ± 6.95	93.48 ± 13.31	97.19 ± 1.65	61.74 ± 7.99	78.73 ± 12.45	93.99 ± 12.18	97.73 ± 1.57
**CPG**	73.65 ± 12.77	99.17 ± 1.06	100 ± 0	99.42 ± 1.3 ^¶^	52.88 ± 17.95	98.84 ± 1.28	100 ± 0	99.49 ± 1.14 ^¶^
**Porcine**	63.32 ± 18.44	94.58 ± 10.31	95.59 ± 7.37	94.66 ± 5.3	65.02 ± 20.42	95.56 ± 8.57	95.98 ± 6.77	95.64 ± 3.91

Results are expressed as percentages of control and are the means ± standard error. * indicates *p* < 0.05 when all groups were better than control within the same timeframe, whilst ^¶^ shows better defect closure for CPG compared to the other groups in the same timeframe (*p* < 0.05).
